# Supplementary material for: Relevance of reporting leprosy related disability at the completion of multi drug therapy: A 5-year retrospective analysis of disability in persons affected by leprosy at ALERT Hospital Ethiopia
Source: PLoS Negl Trop Dis. 2024 Dec 13;18(12):e0012720. doi: 10.1371/journal.pntd.0012720 (PMC11676867; doi:10.1371/journal.pntd.0012720)
Supplement: Attachment — Submitted filename: Leprosy Ethiopia (1).docx [file pntd.0012720.s003.docx]

**Relevance of reporting leprosy related disability at the completion of multi drug therapy:**

**A 5-year retrospective analysis of disability in persons affected by leprosy at ALERT**

**Hospital Ethiopia**

**Key Review Criteria Required for Acceptance?**

As you describe the new analyses required for acceptance, please consider the following:

**Methods**

-Are the objectives of the study clearly articulated with a clear testable hypothesis stated?
-Is the study design appropriate to address the stated objectives?
-Is the population clearly described and appropriate for the hypothesis being tested?
-Is the sample size sufficient to ensure adequate power to address the hypothesis being tested?
-Were correct statistical analysis used to support conclusions?
-Are there concerns about ethical or regulatory requirements being met?

*- Yes*

*- Based on the aim of the study stated at line 83-85, the quantitative method is sufficient for primary objective. I suggest the qualitative method for secondary outcomes*

*- Has the semi structured interview question got trial before? How to prevent bias with those open-ended question method? Line 130-133*

*- sample size is sufficient*

*- statistical analysis is correct for quantitative data, but how you manage the quantitative data for present the results?*

*- no concern about ethical issue*

**Results**

-Does the analysis presented match the analysis plan?
-Are the results clearly and completely presented?
-Are the figures (Tables, Images) of sufficient quality for clarity?

- *Is the information of line 156-170 the result of the study? if not and you want to provide the comparative data, you can state in the discussion section*
- *Line 189-190: Do you mean 197 out of 303 patients who had complete data previously? Or are those 197 from all patients who completed MDT?*
- *Line 178-181: if you also want to give specific data about the child, you should state it at the method*
- *For table 4: how do you count the percentage? Is the absent + present disability equal to the total of the patient?*
- *For the interview result, can you present it in the graphics or something to make more attractive?*

**Conclusions**

-Are the conclusions supported by the data presented?
-Are the limitations of analysis clearly described?
-Do the authors discuss how these data can be helpful to advance our understanding of the topic under study?
-Is public health relevance addressed?

*- Line 403-406: you might complete the statement with the decline of disability data at completion of MDT*

*- The limitation analysis is appropriate*

*- Yes*

*- Yes*

**Editorial and Data Presentation Modifications?**

Use this section for editorial suggestions as well as relatively minor modifications of existing data that would enhance clarity. If the only modifications needed are minor and/or editorial, you may wish to recommend “Minor Revision” or “Accept”. (Limit 20000 Characters)

*-accept with minor revision-*
