# Supplementary material for: Relevance of reporting leprosy related disability at the completion of multi drug therapy: A 5-year retrospective analysis of disability in persons affected by leprosy at ALERT Hospital Ethiopia
Source: PLoS Negl Trop Dis. 2024 Dec 13;18(12):e0012720. doi: 10.1371/journal.pntd.0012720 (PMC11676867; doi:10.1371/journal.pntd.0012720)
Supplement: Attachment — Submitted filename: Editors.docx [file pntd.0012720.s005.docx]

**Dear Journal Editors and Reviewers,**

Thank you very much for reviewing our manuscript submitted to journal of PLOS Neglected Tropical Diseases.

We have gone over the comments and suggestions thoroughly with attention to details and we have included in this letter all the comments with their respective responses and changes made highlighted with yellow for convenience.

**1**.Reference: The references need extensive review.

- *I have done extensive review of all references once again.*

**2.**Figure 1: Figure 1 needs revision; it was cut off.

- *I revised it and added the missing part.*

**3.**Disability Data: The global and WHO regional disability data were presented only as percentages. It is important to also present these data in absolute numbers. Additionally, the authors should discuss the current number of people with disabilities due to leprosy worldwide. If the absolute numbers are decreasing, potential reasons should be explored, including the impact of the COVID-19 pandemic and the poor awareness mentioned in the discussion. The WHO estimates that between 2 and 3 million people are currently living with disabilities due to leprosy, and this should also be discussed.

- *I have included the current WHO estimate of patients living with disability due to leprosy which is 3-4 million people. I have added and cited this point in the introduction part of the revised manuscript in line 77-78.*
- *I have added the absolute numbers besides the percentage values of Grade 2 Disability(G2D). I focused on the percentage of G2D since this is an important indicator for WHO as this indicates how late leprosy cases are diagnosed. The crude actual numbers may not be an ideal indicator to track Grade 2 disability in leprosy as the proportion of G2D among newly diagnosed leprosy cases is more informative.*
- *I have also discussed the effect of COVID-19 pandemic in relation to decline of the reported leprosy cases in the discussion part in line 302-304.*

**4.**Line 206: Please include the dropout rate at ALERT Hospital. It appears that 309 patients were treated there, and 63 received the first dose but did not return. What would the dropout rate be in this case? This is important because more than half of these patients had G2D.

- *63 patients have discontinued treatment while 309 patients had completed MDT at ALERT. Thus, out of the total 372 patients who started treatment and follow-up, the dropout rate was 63/372=16.9% and I have included this in line 193-195 as I thought it would be a better place to put it.*

**5.**Line 215: “Error! Reference source not found.”

- *I have added Table 2 as a cross-reference in line 222.*
- *In addition, I have noticed that a reference should be added in Line 212 and added it.*

**6.**Table 2: More than 50% of the diagnosed patients were skin smear positive. This is very high. Could this be due to late diagnosis? Please discuss.

- *Smear positivity is seen in more than 50% of patients diagnosed at ALERT and this could be due to late diagnosis and poses significant risk of transmission in the community and alarms high index of suspicion. I have included this in the discussion starting from line 342-344.*

**7.**Evaluation of Disability Grades: People evaluated for disability grade at diagnosis: 303; people evaluated for disability grade at completion: 196 (64.7%). The situation is worse in children: 29 to 14 (48.3%). The change in disability may be much worse than depicted. This needs to be part of the discussion starting at line 327, where the low percentage of disability worsening at ALERT is correlated with the high quality of care at ALERT. However, it should be noted, as mentioned in the previous paragraph, that more than half of the patients with G2D discontinued treatment. High-quality care should prevent this. Other possibilities should be discussed.

- *I agree that the change in disability could be much worse than what is shown in the study and could have been underestimated and I have included this in the discussion part mentioning some of the possible reasons starting from line 360-365*
- *I have removed the initial explanation for the low disability worsening at ALERT related to higher quality of care delivered and discussed this could be due to the significant number of patients especially children’s disability grades are not assessed at the end of treatment. Also, problems with proper disability assessment and recording could have contributed.*
- *In addition, since more than half of patients with G2D discontinued treatment, other than what I already mentioned, I have also added issues/problems with the quality of services delivered at ALERT at the end of this paragraph.*

**8.**Line 310: Could you please further discuss the issue of 9.4% of children among those who completed treatment at ALERT? Although 9.4% seems high, in other countries, like the Comoros or some regions of Indonesia, these percentages can exceed 30%. If an active search is implemented, perhaps the percentage of children diagnosed with leprosy would increase. Regarding the other African countries mentioned, is the lower percentage a good or bad indicator of leprosy diagnosis in those countries?

- *I have done some further literature review and childhood leprosy could be even higher in some countries like Comoros where it was 27% in 2016, and I have included this in the discussion part in the line starting 334-336.*
- *Regarding the other African countries mentioned, if proper case surveillance and identification was done, then the low percentage could be a good indicator but if the surveillance system was poor then it could be a bad indicator.*
- *Generally, the WHO uses the percentage of Grade 2 disability in children as a good indicator for active community transmission and if this value is low, it may indicate the national leprosy programmes are doing good assuming the case surveillance is being done properly.*

**9.**Repetitive Citations: It is not necessary to repeat the citation of tables and/or figures during the discussion.
 *I have removed all the repeated table and figure citations in the discussion.*

Reviewer #2:

**10.** Has the semi structured interview question got trial before? How to prevent bias with those open-ended question method? Line 130-133

- *Pilot testing was done to identify any unintended leading questions, ambiguous phrasing or interviewer bias.*
- *To avoid bias especially with the open-ended questions, we used a standardized set of core questions ensuring that all participants were asked about the same topics. This enables to reduce bias by limiting deviations from the main topic of interest.*
- *I have addressed the above issues in line 137-138.*

***11.*** statistical analysis is correct for quantitative data, but how you manage the quantitative data for present the results?

- *We managed the quantitative data using the ODK and checked for completeness and any error at daily basis and data was stored on*

*LSHTM Server.*

*(I hope the question is about quantitative not qualitative data)*

Reviewer #1:

**12**.The analysis is well-presented and matches the plan. I had one minor note about Table 1: It should be specified that the first numbers are actual N/numbers. At the bottom it just says, “Data are number (%) unless otherwise specified.”

*Okey, noted. I have rephrased it as “Data are in the order of numbers(N) followed by percentage”*

 Reviewer #2:

**13**. Is the information of line 156-170 the result of the study? if not and you want to provide the comparative data, you can state in the discussion section

- *Actually, it is part of the study. Analysis of published WHO leprosy related Grade 2 disability(G2D) globally, regionally and in Ethiopia using the Weekly Epidemiological Record (WER) was done and this was mentioned on the methodology part and the presented analysis is part of the study.*

    **14**.Line 189-190: Do you mean 197 out of 303 patients who had complete data previously? Or are those 197 from all patients who completed MDT?

- *It means 197 out of the 303 patients with complete disability data at diagnosis(initially) and completed MDT, when evaluated at the end of MDT didn’t have disability data recorded. I already used the linking phrase “of them” on the manuscript on line 196 of the revised manuscript.*

**15.** Line 178-181: if you also want to give specific data about the child, you should state it at the method

- *I have now mentioned this in the method section in line 92 in the new manuscript.*

**16.** For table 4: how do you count the percentage? Is the absent + present disability equal to the total of the patient?

- *Sorry for the confusion here. The absent +present disability equals to the total number of patients diagnosed with leprosy at ALERT and have complete disability data at diagnosis. i.e. the 303(98%) of patients diagnosed at ALERT.*
- *I have modified table 4 labelling to make to make it more clear.*

**17.** For the interview result, can you present it in the graphics or something to make more attractive?

*Okey, I have inserted a new figure summarizing the interview results starting from line 278-289 in the new revised manuscript.*

**18.**-Line 403-406: you might complete the statement with the decline of disability data at completion of MDT

- *Okey, I agree. I have included this in the conclusion part in line 436.*

Reviewer #1:

Minor editorial notes:

 **19**. Line 39: enragement—maybe “enlargement”?

- *Sure, it is enlargement. I have corrected it.*

**20**.Lines 75-79: I think there is a word or two missing here. I think maybe “to” needs to go before “estimate,” or you could say “for early case detection and estimation of the delay”

- *Okey, great. I have used the later suggestion and rephrased in line 80 of the revised manuscript.*

**21**.Line 138: “on daily basis”—should be “on a daily basis”

- *Agree, I have corrected it in line 140-141 of the revised manuscript.*

**22**.Line 160: “Eastern Mediterranean”—I see that WHO includes 22 countries (including parts of North African and the Horn of Africa) in their Regional Office for the Eastern Mediterranean but not Ethiopia. You might give more information here about how Eastern Mediterranean is defined though.

- *Okey, I have included about the definition of Eastern Mediterranean definition from the WHO website in line 163-165.*

**23**.Line 162: “increment” –should this be “increase” or maybe “incremental change”?

- *Okey Agree, I have used the term “increase” in line 167 of the revised manuscript.*

**24**.Line 178: “child” should be “children”

- *Sure, I agree. I have corrected it in line 183 of the revised manuscript.*

**25**.Line 215: “reference source not found” –should be resolved here or deleted?

- *I have added Table 2 as a cross-reference in line 222.*
- *In addition, I have noticed that a reference should be added in Line 212 and added it.*

**26.**Line 338: “older” –should be just “age” or “being older”

*I agree. I have used the term “being older” in line 368 of the revised manuscript.*

 **27.**I have included minor editorial notes in a box above. My only other suggestion is that you elaborate more on leprosy reaction. You mention that some healthcare workers talked about patients discontinuing medications when they develop leprosy reactions. You could have a brief explanation of what leprosy reactions are in the section on clinical aspects of leprosy, and then I think it is important to highlight how easy it is for patients to misunderstand what is happening when they develop symptoms of leprosy reaction after starting or while taking MDT, especially if HCWs have not really explained this to them in advance. This could also be mentioned briefly in the discussion section as well and of course also related to the importance of training for all HCWs working with people affected by leprosy.

- *I have further elaborated about clinical features of leprosy reactions in line 51-58.*
- *I have mentioned about the importance manging leprosy reactions in the discussion part.*
- *In the recommendation part, I have mentioned about training health care workers about managing leprosy reactions and proper patient counselling in line 456-457*

**NOTE**: Please note that all the line numbers I mentioned in this document corresponds to the final revised document **without track changes.**

There might be a few lines distortion in the line number of the revised document with track changes.

Kind regards,

Bereket Abebayehu Tegene M.D, MSc, DTM&H

<Tel:+251916109982>

Email: abebayehu.bereket25@gmail.com
